# Supplementary material for: An experimentally-informed polymer model reveals high resolution organization of genomic loci
Source: Nat Commun. 2026 Feb 4;17:2338. doi: 10.1038/s41467-026-68928-w (PMC12979793; doi:10.1038/s41467-026-68928-w)
Supplement: Supplementary file 1 — Supplementary Information [file 41467_2026_68928_MOESM1_ESM.pdf]

Supplementary Information for
An experimentally-informed polymer model reveals high resolution organization of genomic loci

Rahul Mittal[1,], **Dieter W. Heermann**[2], **and Arnab Bhattacherjee**[1,2,*]

[1]School of Computational & Integrative Sciences, Jawaharlal Nehru University, New Delhi, 110067, Delhi, India
[2]Institute for Theoretical Physics, Heidelberg University, Philosophenweg 19, Heidelberg, 69120, Heidelberg, Germany.
[*]arnab@jnu.ac.in

Supplementary Methods

Detailed method

We develop a simplified multi-scale polymer model to understand the chromatin organization at different resolutions. Our model aims to simulate a 0.2 Mb long chromatin segment. We use Hi-C contact map information at 5 kb resolution to capture global organization. The Hi-C contact map tells about the average contact frequency between different genomic segments among the population of cells. So, we distribute the Hi-C contact information among 100 different conformations of a homo-polymer chain with 40 beads, each representing a 5 kb segment of the target region. Important contacts are distributed among these conformations. A contact is known as an important contact if the contact frequency between any i, j (belonging to 40 beads), is higher than the average frequency($|j-i|$)+ standard deviation of the distribution of frequency($|j-i|$)[1].

$$P_{ij} = \begin{cases} 1, & f_{avg}(|j-i|) \pm f_{std}(|j-i|) < p_{ij} \\ 0, & \text{otherwise.} \end{cases} \tag{1}$$

Here $f_{avg}(|j-i|)$ is the average contact frequency for the genomic segment having the same |j-i| for every i,j having contact probability p_{ij} and $f_{std}(|j-i|)$ stand for standard deviation. The contacts are distributed according to the gaussian probability distribution of contacts among the conformation. For each conformations, we get the contact information to be in contact for each genomic segment bead pair (i,j) as binary matrix. These matrices are used to restrict the dynamics of our simulation for high-resolution copolymer chains representing the target region. The contact information is not enough to generate an ensemble average contact that mimics the Hi-C contact map because dynamics play a major role in manipulating the contact frequency of the simulated ensemble average contact map. Before using this contact information directly we simulate these 100 conformations using a homopolymer chain (Supplementary Figure 1) of 40 beads representing the 0.2 Mb segment where each bead represents a 5 kb region. The binary matrix holds the contact information for |j-i| more than 1 as in a polymer chain all the adjacent beads remain always in contact. This helps to know whether the dynamics favour mimicking our global-level organization.

$$U^{H}_{Total} = U^{H}_{bond} + U^{H}_{HICcont} + U^{H}_{WCA} \tag{2}$$

$$\frac{U^{H}_{WCA}(r_{ij})}{k_B T} = \begin{cases} 4\left[\left(\frac{d_{ij}}{r_{ij}}\right)^{12} - \left(\frac{d_{ij}}{r_{ij}}\right)^{6}\right] + 1\ ; & r_{ij} < 2^{\frac{1}{6}} d_{ij} \\ 0, & \text{otherwise.} \end{cases} \tag{3}$$

$$U^{H}_{bond}(r_{ij}) = K_{harmo}(r_{ij} - r_0)^2\ ;\ |j-i| = 1\ ;\ r_0 = 30nm \tag{4}$$

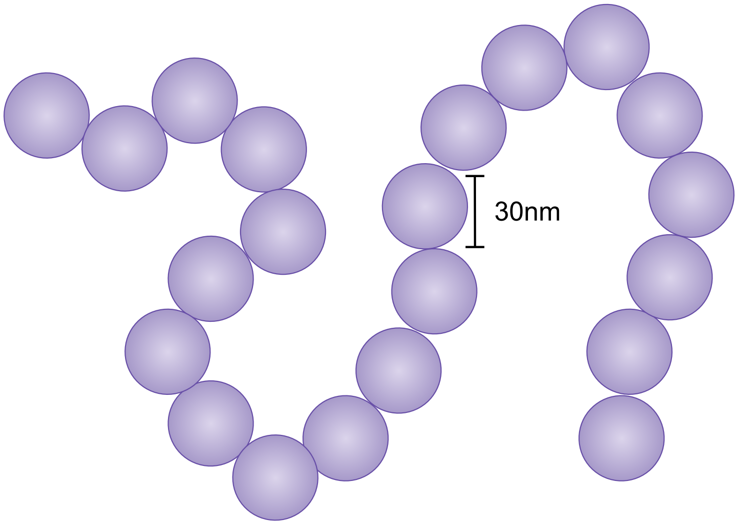

Supplementary Figure 1. Schematic diagram of a homopolymer chain at 5 kbp resolution with a diameter of 30nm.

$$U^{H}_{HICcont}(r_{ij}) = K_{hic}(r_{ij} - r_0)^2 \; ; \; |j-i| >= 2 \; \& \; P_{ij} = 1 \; ; \; r_0 = 30nm \tag{5}$$

In WCA potential, d_{ij} is the average diameter of interacting particles, has the value of 30nm and K_{harmo} is the strength for the adjacent beads to be in contact with the value $100k_BT/nm^2$. We systematically allow contact from a binary matrix to be formed taking care of unwanted chaos. We start from a closer genomic segment (|j-i|) to be in contact with the binary matrix and move in ascending order of |j-i| as the previous pair complete the bond formation. Once all the binary contact is established, we start to dump the structure and calculate the ensemble average contact map. After getting a good agreement with the Hi-C contact map we start our high-resolution simulation with the copolymer chain. The copolymer chain has two types of beads (Supplementary Figure 2) that differ in size, the bigger ones represent nucleosome bead and the smaller ones represent linker bead. Nucleosome beads represent 142 base pairs, and the linker DNA bead represents around 8 base pairs. The position of the nucleosome beads in the copolymer chain is decided DANPOS[2] software. In MNase-Seq, micrococcal nuclease (MNase) preferentially digests linker DNA, leaving behind nucleosome-protected DNA fragments that are aligned to the genome. DANPOS then processes the aligned reads by smoothing coverage profiles, identifying statistically significant peaks, and estimating dyad positions and occupancy scores. This yields a genome-wide map of the most probable nucleosome positions, which reflect consensus (population-averaged) nucleosome locations across many cells. These high-confidence dyad positions are used in our model to place nucleosome beads along the polymer chain.

The polymer chain at near base pair resolution has a larger number of beads to simulate and cause computational complexity. Further, to overcome this complexity we develop a GPU-based simulator using CudaC programming language with cuda version 11.0 and GeForce GTX10 architecture. This simulator provides 25× speedup than a based simulator to handle around 50,000 beads system. Using the simulator, we simulate our high-resolution copolymer chain of around 10,000 NL beads representing 0.2 Mb chromatin segment. We use Langevin dynamics to simulate our system using a simplified polymer model where every ith bead has position r_i in the polymer chain and r_{ij} is the distance between ith and jth particle. The system has the total energy:

$$U_{Total} = U_{FENE} + U_{WCA} + U_{FENE} + U^{COM}_{WCA} + U^{COM}_{HICcont} \tag{6}$$

The consecutive beads are connected by the finite extensible nonlinear elastic (FENE) potential:

$$U_{FENE}(r_{i,i+1}) = U_{WCA}(r_{i,i+1}) - \frac{K_{FENE}R_0^2}{2} \log\left[1 - \left(\frac{r_{i,i+1}}{R_0}\right)^2\right] \tag{7}$$

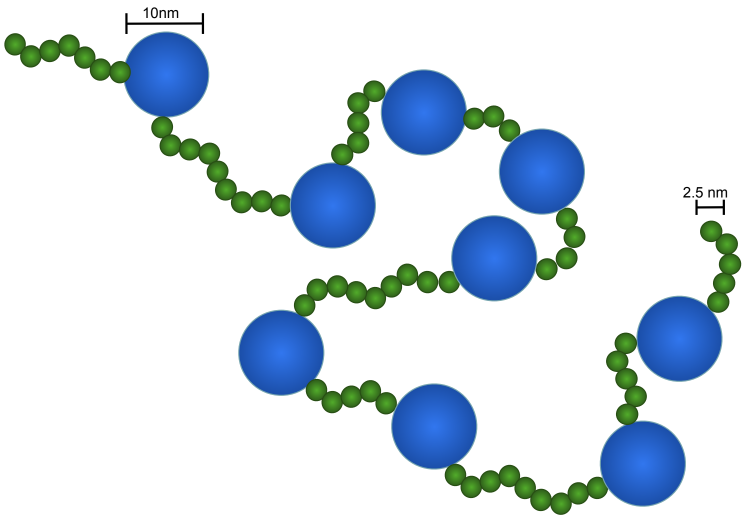

Supplementary Figure 2. Schematic diagram of copolymer chain (representation of NL beads) with two types of beads; smaller size bead has the diameter of 2.5nm and larger size bead has the diameter of 10nm.

Also, consecutive three linker beads are linked using Kratky-Porod potential, which helps them to maintain the persistent length between linker DNA beads. The magnitude of the bending potential manipulates the persistence of linker DNA.

$$U_{BEND}(\theta) = K_{BEND}\left[1-\cos(\theta)\right] \tag{8}$$

where,

$$\cos(\theta) = (r_i - r_{i-1}).(r_{i+1} - r_i) \tag{9}$$

Here θ is the angle formed by three consecutive linker DNA beads and $K_{BEND} = l_p k_B T$ for linker DNA beads. l_p is the persistence length considering 147 linker base pairs to behave like a rod, resulting in a length of around 50nm. We use σ to represent the size of the linker DNA bead. In the unit of σ, l_p is 20σ. To avoid overlapping between non-bounded beads, we use Weeks-Chandler-Andersen(WCA) potential taking care of all the steric interactions.

$$\frac{U_{WCA}(r_{ij})}{k_B T} = \begin{cases} 4\left[\left(\frac{d_{ij}}{r_{ij}}\right)^{12} - \left(\frac{d_{ij}}{r_{ij}}\right)^{6}\right] + 1, & \frac{r_{ij}}{2.5} < 2^{\frac{1}{6}} d_{ij} \; and \; d_{ij} = 10nm \\ 4\left[\left(\frac{d_{ij}}{r_{ij}}\right)^{12} - \left(\frac{d_{ij}}{r_{ij}}\right)^{6}\right] + 1, & r_{ij} < 2^{\frac{1}{6}} d_{ij} \; and \; d_{ij} \neq 10nm \\ 0, & \text{otherwise.} \end{cases} \tag{10}$$

In our copolymer chain, the nucleosomal bead has a diameter of 10nm and linker DNA bead has a diameter of 2.5nm[3] as each base pair has a size of $0.34nm$. Here $d_{i,j}$ refers to the average diameter of interacting beads and $r_{i,j}$ is the distance between r_i and r_j. In eq. (7) is R_0 is $1.6d_{i,j}$. The copolymer chain simulations beads, which provide a high-resolution perspective of the homopolymer chain bead, are restricted using binary contact map information(distributed HiC contact map information using homopolymer chain simulation). Initially, we identify the NL beads, which represent the 5 kb genomic segment, related to homopolymer chain beads. Then we constrain the motion of NL beads with respect to the interacting 5 kb genomic segment pair. We consider two 5 kb genomic segments to be in contact based on a binary contact map. If two 5 kb genomic segments are in contact, they maintain the reference distance between 5 kb genomic regions, where the position of each genomic region is identified as the center of mass position of the involved NL beads. If two 5 kb genomic segments are not in contact, they follow the excluded volume potential to avoid segmental overlapping. Each 5 kb region has a large spherical space of diameter 90nm, following the base-pair density[4] in the nucleus, to provide a required entropic environment for NL beads to maintain the density.

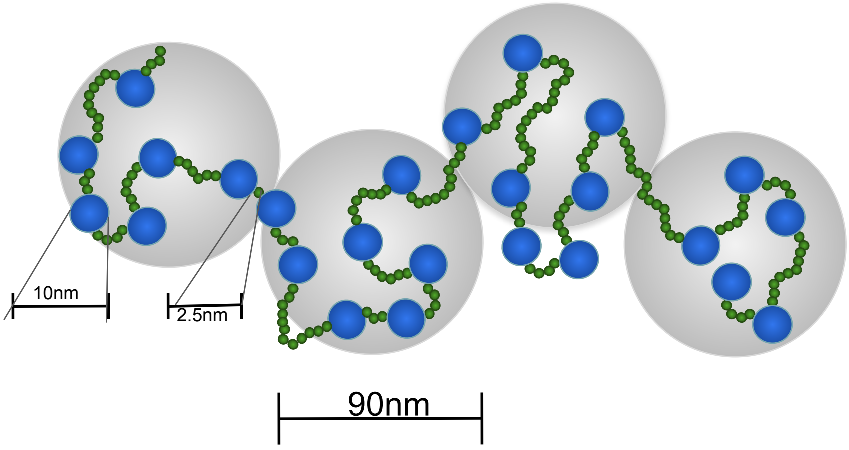

Supplementary Figure 3. Schematic diagram of NL beads constrained contact information; two interacting 5 kbp genomic segments maintain a center of mass distance of 90nm.

$$\frac{U_{WCA}^{COM}(r_{ij}^{COM})}{k_B T} = \begin{cases} 4\left[\left(\frac{r_0^{COM}}{r_{ij}^{COM}}\right)^{12} - \left(\frac{r_0^{COM}}{r_{ij}^{COM}}\right)^{6}\right] + 1\ ;\ r_{ij}^{COM} < 2^{\frac{1}{6}} r_0^{COM} \\ 0,\ \text{otherwise.} \end{cases} \tag{11}$$

$$U_{HICcont}^{COM}(r_{ij}^{COM}) = K_{hic}^{COM}(r_{ij}^{COM} - r_0^{COM})^2\ ;\ |j-i| >= 2\ \&\ P_{ij} = 1\ ;\ r_0^{COM} = 90nm \tag{12}$$

During the simulation, the position of beads is updated by following the Langevin equation

$$m_i \frac{d^2\mathbf{r}_i}{dt^2} = -\nabla U_i - \gamma_i \frac{d\mathbf{r}_i}{dt} + \sqrt{2k_B T \gamma_i}\eta_i(t) \tag{13}$$

Here m_i is mass of respective bead, r_i is the position vector, γ_i is the fraction felt due to implicit solvent and $\eta_i(t)$ is uncorrelated random noise that follow the equation mentioned below

$$\langle \eta_\alpha(t) \rangle = 0 \tag{14}$$

$$\langle \eta_\alpha(t)\eta_\beta(t^{'}) \rangle = \delta_{\alpha\beta}\delta(t - t^{'}) \tag{15}$$

The noise is controlled by thermal fluctuation in the form of thermal energy as a multiplication of Boltzmann coefficient (k_B) and temperature (T). Here with the reduced unit parameter, we use T=1. The U_i is the summation of all the potential representing the polymer chain interaction. Here we consider the unit mass and friction coefficient for all the beads in the

reduced unit. To solve the above equation we use the velocity-Verlet algorithm with the time step $\Delta t = 0.005\tau$ where τ is the simulation time unit. We initialize the simulation with a linear chain. We provide inter nucleosome potential using eq.(11) and the contact information we distributed in 100 conformation from Hi-C contact map put constrain among NL beads to follow the connection between 5 kb regions. We simulate for 50000 τ and after 17500 τ we start to dump the structure for every 250 τ. Our system satisfies the restriction imposed to capture global organization details within initial 17500 τ. We have mentioned initially that we distribute the HiC contact map information in 100 binary matrices at 5 kb resolution using a homopolymer chain. Each binary matrix is used as a constraint for high-resolution copolymer chain simulation, where a set of beads in a copolymer chain represents a homopolymer chain bead (region of 5 kb). To capture the high-resolution contact map for the homopolymer chain with a resolution of 5 kb, we simulate 100 simulations, constrained by each of the 100 binary matrices, for a 0.2 Mb system. Throughout the simulation, we get 300 conformations and select 130 conformations, discarding the initial 170 conformations. We end up with 13000 conformations after simulating the system for all the restriction layouts.

Distance Corrected Pearson Correlation Coefficient

We note that distance-corrected correlation coefficients become unstable for small matrices (0.2 Mb at 5 kb resolution yields only 40 bins per side, i.e. <40 diagonals). The relatively low dcPCC values (0.5) should therefore be interpreted with caution, as they reflect limited sample size rather than poor structural agreement. At 200 bp resolution, where matrices are large, dcPCC[5] shows strong concordance with experiment across loci (Supplementary Table 1).

Supplementary Table 1. Distance Corrected Pearson correlation coefficient (dcPCC) at 200 base-pair resolution to compare the experimental and simulated contact map for all four genomic loci

System	HoxA13	HoxB4	Lin28A	Nanog
dcPCC	0.74	0.76	0.71	0.80

Supplementary Table 2. Comparison of Spearman correlation coefficient (SCC) and Pearson correlation coefficient (PCC) between simulated and experimental maps represented by an n*n matrix at 5 kb (n=40) and 200 bp (n=1000) for five 0.2 Mb regions. Higher correlation values indicate better agreement with experimental data.

Target Region	27.07-27.27Mb chr7	7.75-7.95Mb Nanog(chr12)	48.5-48.7Mb HoxB4(chr17)	26.32-26.52Mb Lin28A(chr1)	27.08-27.28Mb HoxA13(chr7)
SCC(5 kb)	0.911	0.851	0.927	0.919	0.924
SCC(200 bp)	0.911	0.931	0.876	0.958	0.877
PCC(5 kb)	0.979	0.971	0.978	0.985	0.977
PCC(200 bp)	0.802	0.774	0.845	0.756	0.792

Experimental data for modelling and validation

We export the Hi-C contactmap[6] using [7] database. We use experimental set 4DNES2M5JIGV with sample H1-hESC (Tier 1) from experimental type in situ Hi-C. Similarly,[6] from experiment set 4DNES21D8SP8 we get micro-C contact map for the same sample for experimental type. We get the nucleosome position data for our target system from nucmap database that provide genome-wide position map of nucleosomes for different species. For H1 cell line with experiment component GSM1194220 for sample id hsNuc0070101 we get the position of nucleosomes for our target region.

ChromHMM

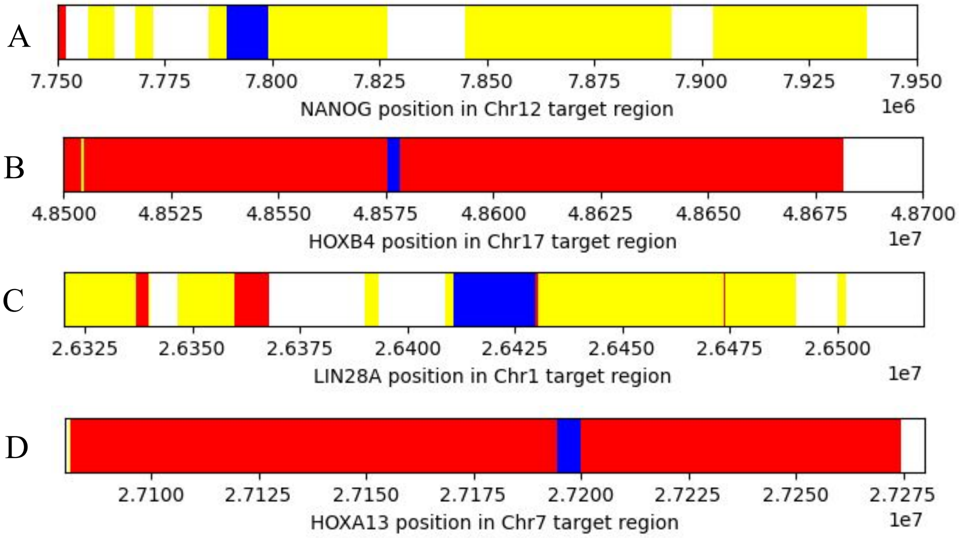

Supplementary Figure 4. Transcriptional activity is identified using ChromHMM for four gene loci A) Nanog, B) Lin28A, C) HoxB4 and D) HoxA13. The red colour shows the transcriptionally inactive region, the yellow colour shows the transcriptionally active region, the blue colour shows the position of the gene position and the uncoloured space shows the undefined transcriptional activity status.

We aim to understand the chromatin organization at different resolutions for a eukaryotic chromatin region. Initially, we simulate for the chr7(27.15-27.19Mb) genomic segment of region 0.2 Mb region. To advance this exploration, we selected 0.2 Mb segments of the Nanog, HoxB4, Lin28A and HoxA13 genomic loci on chromosomes 12, 17, 1 and 7, respectively, in human embryonic stem cells (hESCs). The rationale for this selection lies in the markedly different transcriptional activities of Supplementary Figure 4A,C and Supplementary Figure 4B,D within hESC cell line, as evidenced by ChromHMM[8] analysis.

ChromHMM-conditioned contact enrichment and effective χ parameter

We classified nucleosomes as "active" (A) or "inactive" (B) using ChromHMM annotations of the loci. For each simulated ensemble, all nucleosome pairs with center–center distances below the cutoff r_c (2.5 $\times$ nucleosome diameter, consistent with Micro-C) were counted. We then calculated a log-odds enrichment score, $E = \ln \frac{N_{AA} \cdot N_{BB}}{N_{AB}^2}$, where N_{AA}, N_{BB}, N_{AB} are the numbers of A–A, B–B, and A–B contacts, respectively. A positive enrichment corresponds to preferential like–like interactions. From this, we defined a segregation index that summarizes the degree of A/B partitioning across the locus.

The observation of E > 0 indicates effective micro-segregation of chromatin states. Following recent theoretical work and data-driven inversion studies, this enrichment can be interpreted as a positive effective Flory–Huggins parameter $\chi > 0$, consistent with the idea that Hi-C–derived effective interactions naturally satisfy Flory–Huggins–like criteria and give rise to A/B compartmentalization. These results situate our findings within the broader framework of polymer-based descriptions of compartment formation.

Supplementary Table 3. χ values for four target genomic loci

System	HoxA13	HoxB4	Lin28A	Nanog
χ	0.960	0.832	0.874	0.840

Loop detection using HiCExplorer[9,10]

Chromatin loops are an essential factor in the structural organization of the genome. To detect the possible number of loops from the MicroC contact map, we used HiCExplorer software, which uses a chromatin loop detection algorithm that applies a strict candidate selection based on continuous negative binomial distributions and performs a Wilcoxon rank-sum test to detect enriched Micro-C interactions. Using HiCExplorer, we calculated the number of loops in our target gene loci. We get a maximum of 5 loops in HoxA13, 4 loops in HoxB4, 3 in Lin28A and no loops Nanog gene loci.

Contact map and boundary prediction of domains

We simulate four different genomic loci of size 0.2 Mb around different genes Nanog, HoxB4, Lin28A and HoxA13. As a simulation result, we get 13,000 structures for each system. We calculate the ensemble average contact map using these structures. To capture the contact map, we coarse-grain the high-resolution copolymer chain into a desired resolution homopolymer chain. To calculate a contact up for the respective resolution we randomly select a genomic pair to be in contact for a random structure and if a random number is smaller than $exp(-\frac{r_{ij}^2}{r_{cut}^2})$ then pair is considered to be in contact. Here r_{ij} is the distance between the i and j beads and r_{cut} is the threshold value to be in contact, considered as 1.2 times of dimeter of the beads. The contact map gives the interaction frequency for each pair of genomic regions and forms domain-like structures along the diagonal of the map. To predict the boundaries of these domains we use slide box algorithm[3,11]. In this method, we sum interaction frequencies of unique pairs in a range and calculate the contact score for each position.

$$score_k = \frac{1}{2d} \sum_{i=k-d+1}^{k} \sum_{j=k+1}^{k+d} x_{ij} \qquad for \quad d < k < N - d \tag{16}$$

After getting a score, we compare the score of the specific position k to $k+K$ upstream and $k+K$ downstream positions scores. If the the target position has the lowest score then it is caller a boundary. The value of k,d and K are chosen based on contact map visualisation that fit better for the contact map domains. The method helps us to capture the domain boundaries(TADs). Further, the method is used to calculate the boundaries for the sub-domain, which can be seen in the main text.

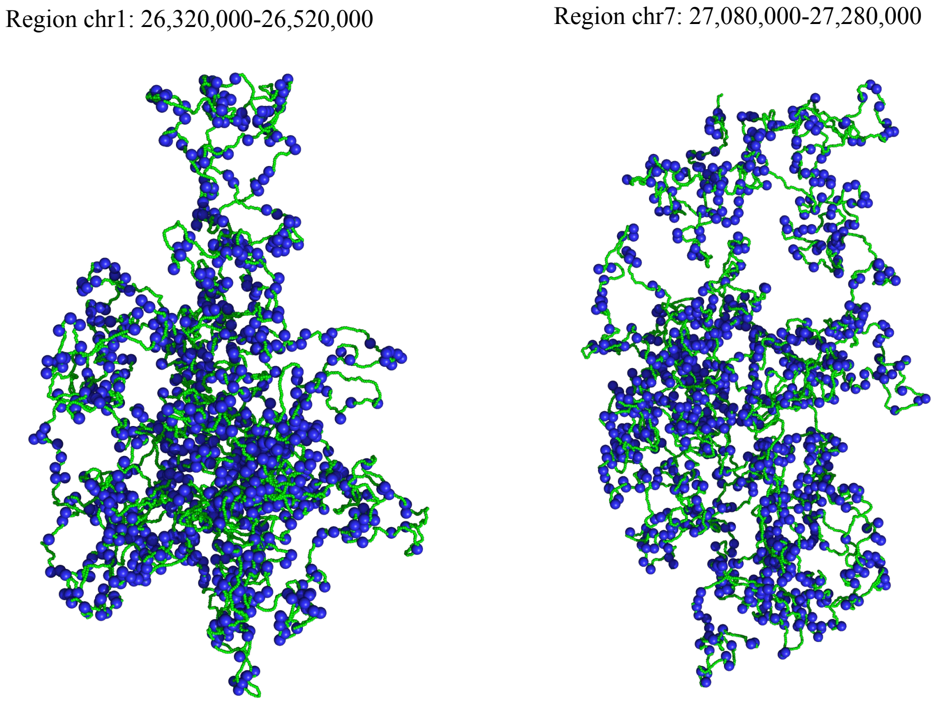

Supplementary Figure 5. Illustrate representative conformations of A) chr1 and B) chr7 chromatin segments around the gene Lin28A and HoxA13, respectively, in hESCs.

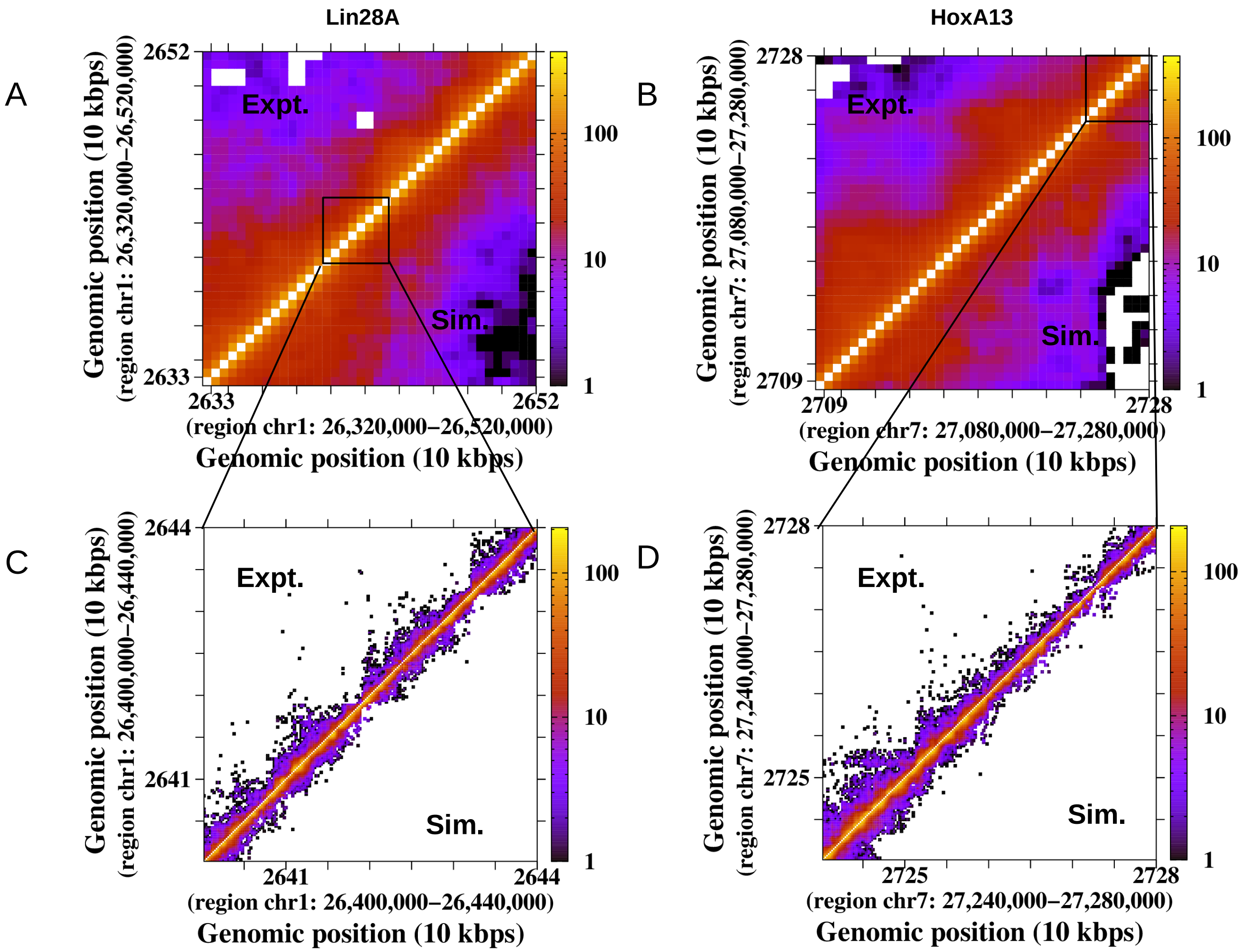

Supplementary Figure 6. Comparison between experimental and simulated contact maps for different chromatin segments of hESCs. (A–B) show the agreement between Hi-C and simulated contact maps at 5 kb resolution for 0.2 Mb chromatin segments from human chromosomes 1 and 7, respectively. (C-D) Highlight the similarities between Micro-C and simulated contact maps at 200 bp resolution for randomly selected 40 kb regions within the same chromatin segments.

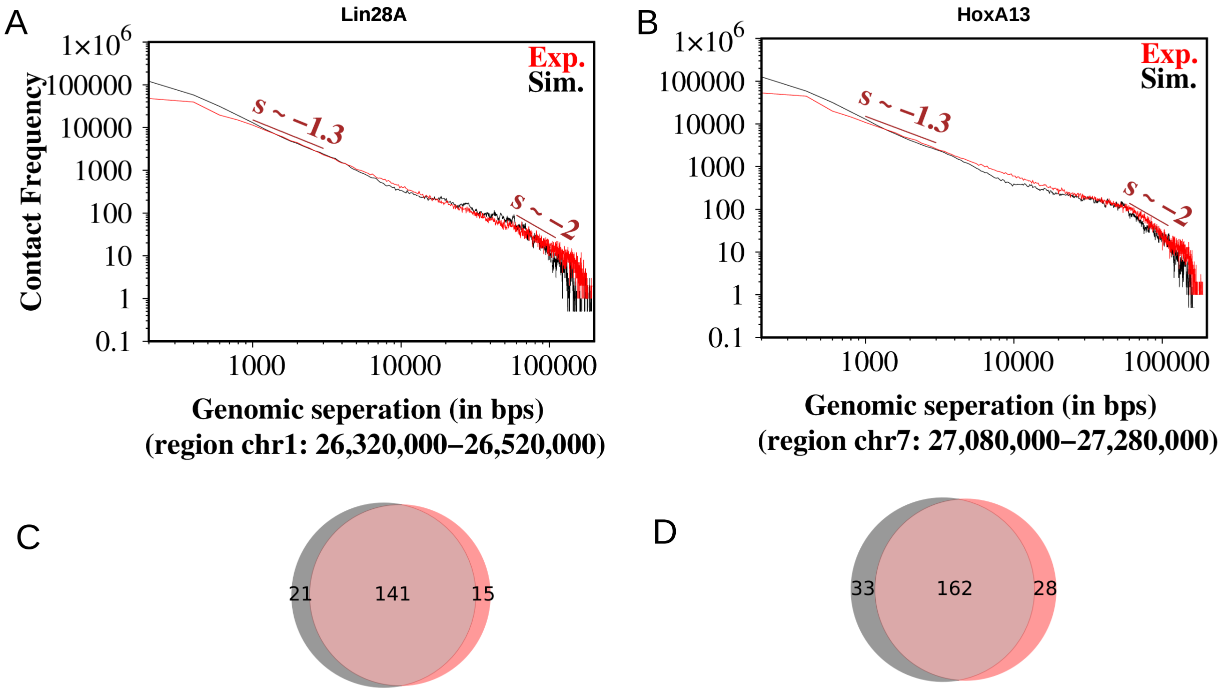

Supplementary Figure 7. Micro-C contact frequencies as a function of genomic separation and prediction of domain boundaries. (A, B) depict the agreement between variations in Micro-C and simulated contact frequencies at 200 bp resolution as a function of genomic separation for three chromatin segments of hESCs. The measured slopes reveal distinct patterns in short-to-intermediate-range and long-range contacts within the Micro-C data. (C, D) show Venn diagrams comparing the predicted domain boundaries identified in Micro-C (red) and simulated contact maps (gray) at 200 bp resolution, highlighting their overlap and differences.

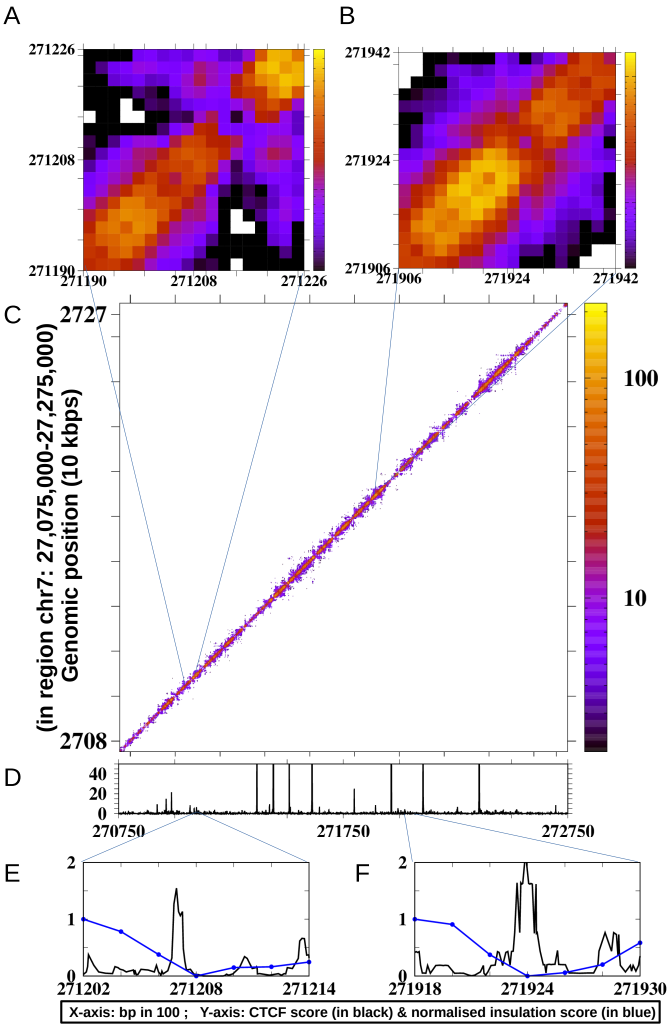

Supplementary Figure 8. CTCF/cohesin binding and insulation profile at a representative model-predicted sub-boundary. Shown are the experimental CTCF ChIP-seq signal (black), normalized insulation score (blue) in Fig. D, and the corresponding Hi-C contact matrix for the 0.2 Mb segment of chromosome 7 in Fig. C. The additional boundary predicted by our model coincides with a local CTCF enrichment peak and a shallow insulation valley in Fig. E, F, supporting its interpretation as a weak but genuine sub-boundary rather than an algorithmic artifact. Fig. A, B show the contact map around the local boundaries shown in Fig. E, F.

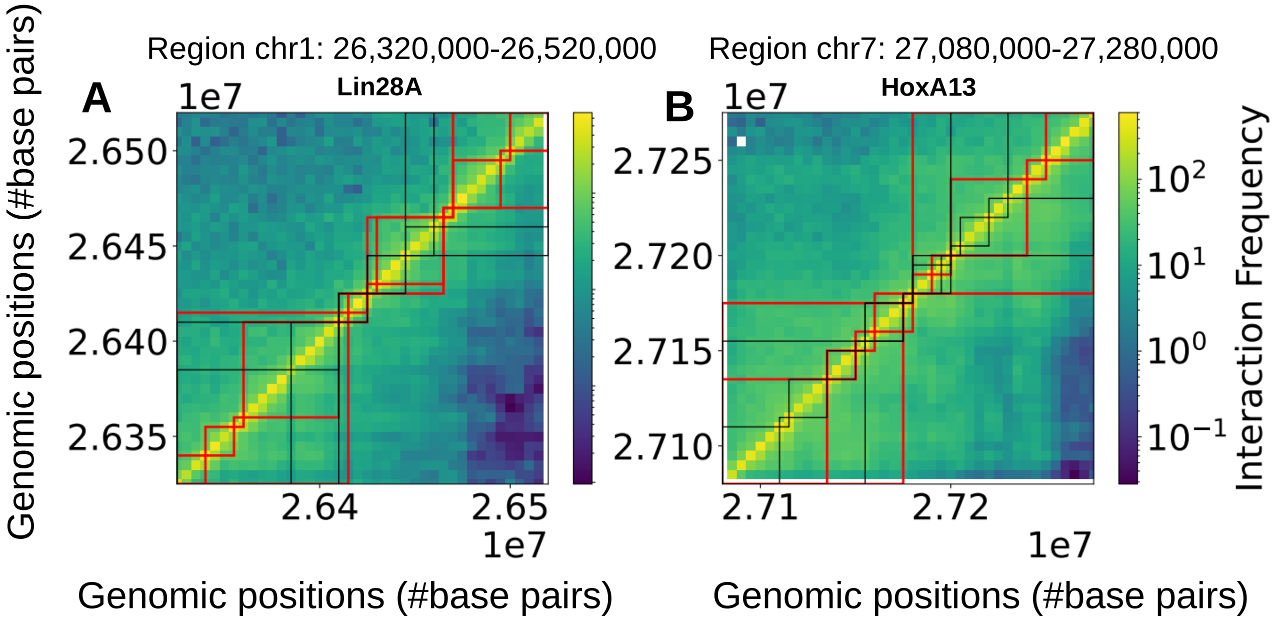

Supplementary Figure 9. Comparison of experimental and simulated contact maps across two genomic regions. (A) Contact map for the Lin28A locus (chr1: 26,320,000–26,520,000). Red boxes mark interaction domains identified from experimental Hi-C data, while black boxes indicate domains detected from simulated contact maps. (B) Contact map for the HoxA13 locus (chr7: 27,080,000–27,280,000), showing similar TAD-like structures. The correspondence between red (experimental) and black (simulation) domains demonstrates that the model recapitulates major interaction features observed in vivo.

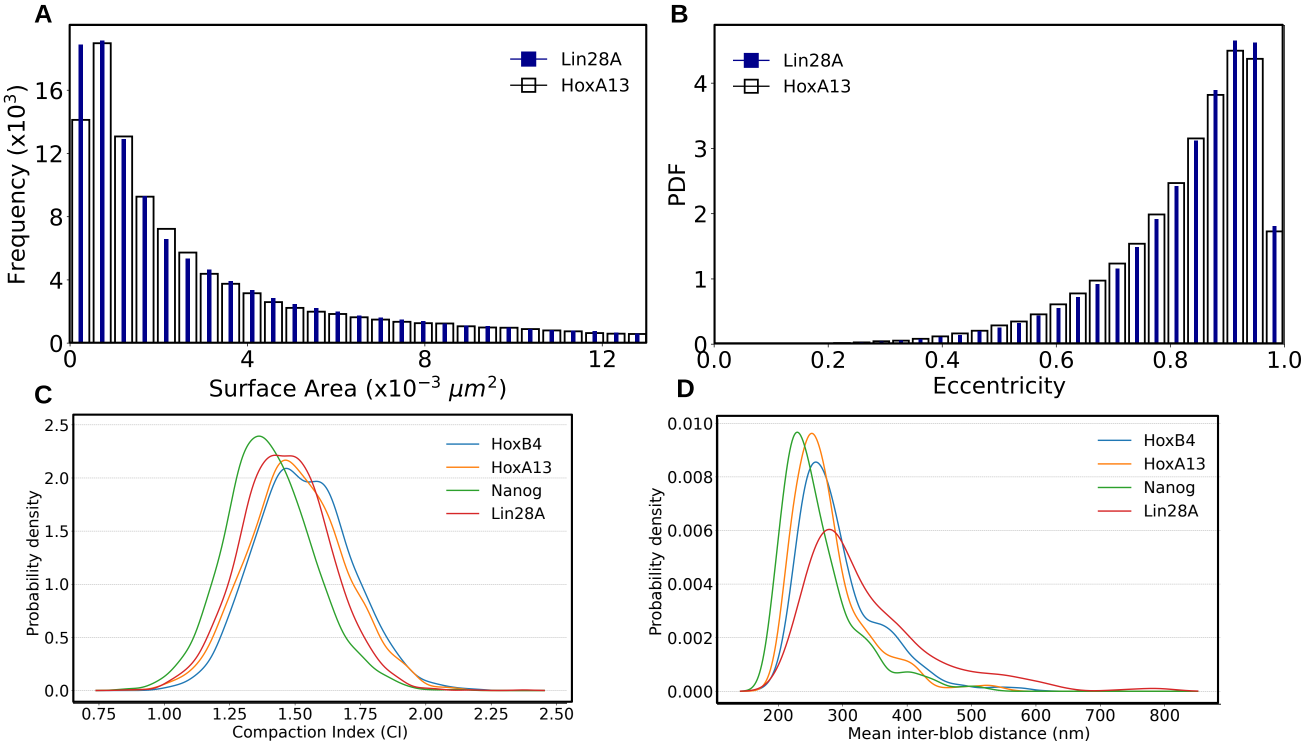

Supplementary Figure 10. Characterizing the morphology of nucleosome blobs. A total of 13,000 NL-level chromatin conformations were analyzed to identify nucleosome blobs for both the Lin28A (filled blue bars) and HoxA13 (open black bars) genomic loci. (A) shows the surface area distribution of blobs, estimated using the convex hull method across all blobs detected by DBSCAN. The results exhibit a lognormal fit with parameters $6.4\times10^{-3}\pm0.015\,\mu m^2$ for Lin28A blobs and $6.7\times10^{-3}\pm0.017\,\mu m^2$ for HoxA13 blobs. (B) presents the eccentricity profile of the blobs, revealing their predominantly ellipsoidal shape. Representative snapshots of nucleosome blobs are shown in the inset. (C) depicts the compaction of blobs in the form of compaction index for all four genomic loci Nanog, HoxB4, Lin28A and HoxA13 loci. (D) illustrates the spatial distribution of nucleosomal blobs by estimating the radial distribution function (RDF) for all four genomic loci Nanog, HoxB4, Lin28A and HoxA13 loci.

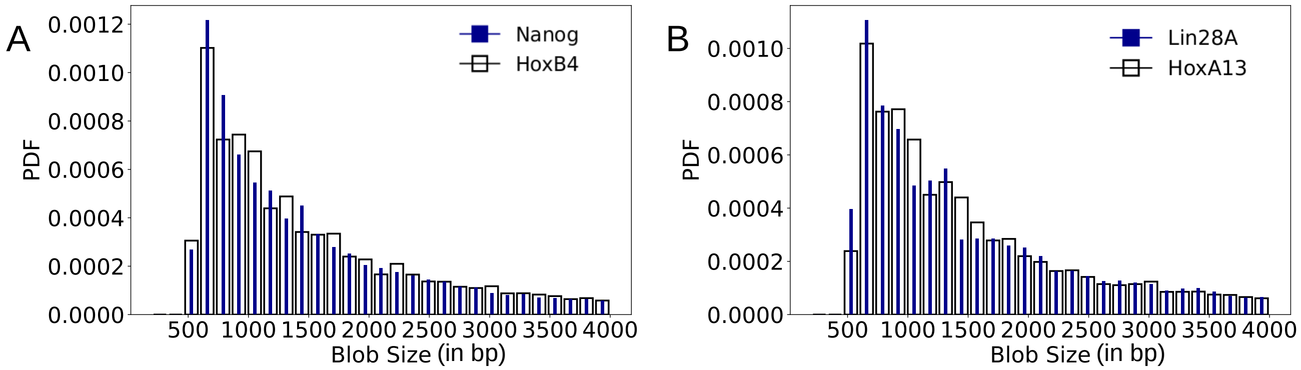

Supplementary Figure 11. Histogram of different blob sizes in a set of active and inactive genomic regions. A) Shows the comparison between Nanog (filled blue bars) and HoxB4(open black bars), and B) shows the comparison between Lin28A (filled blue bars) and HoxA13(open black bars). The median value for the blob size is around $\sim 600-700$ bps.

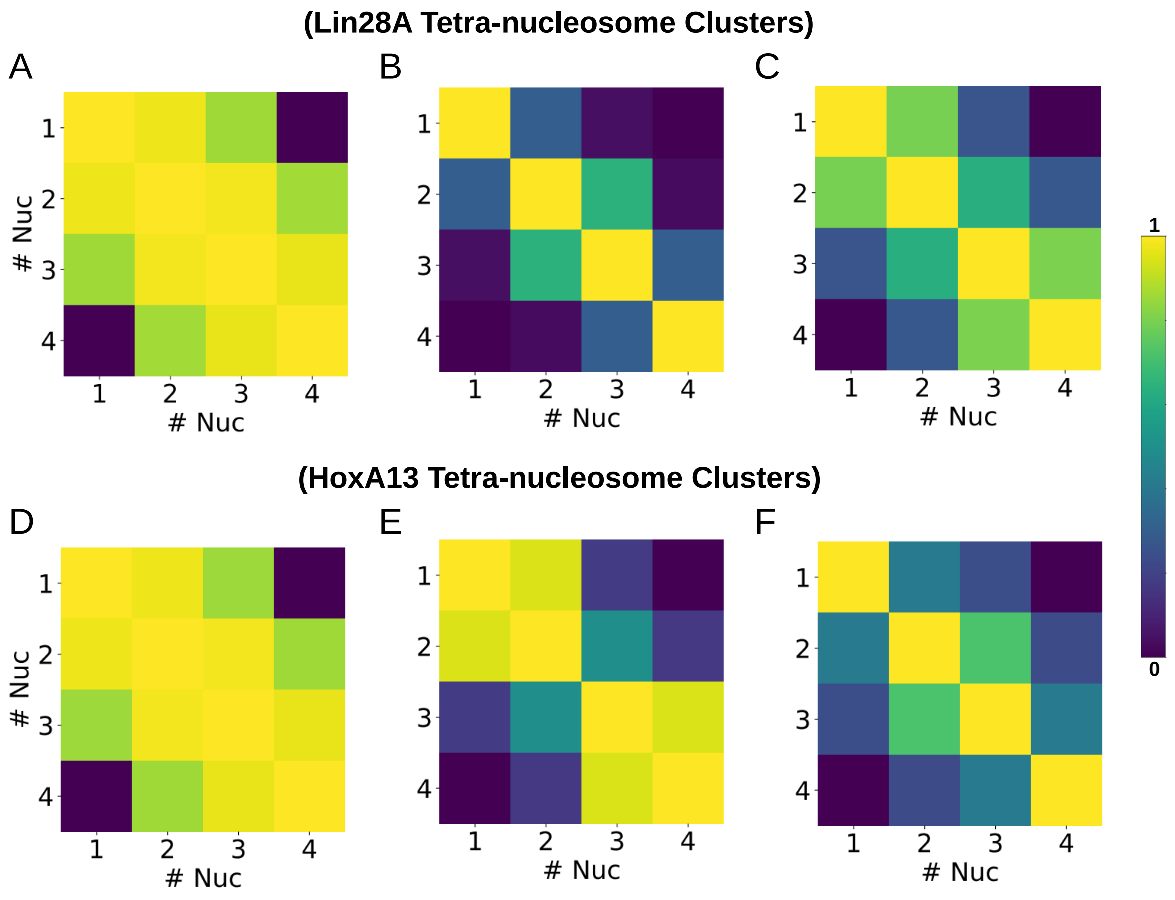

Supplementary Figure 12. Tetra-nucleosome contact maps of Lin28A and HoxA13 genomic loci. An ensemble of tetra-nucleosome contact maps was generated by considering all consecutive sets of four nucleosomes and identifying whether they form contacts. A contact was defined when the pairwise distance between two nucleosomes was within a cutoff distance r_c, set as 2.5 times the nucleosome diameter. The k-means clustering algorithm was employed to identify the top three dominant clusters for both Lin28A and HoxA13 genomic loci. Notably, these three clusters were the only ones comprising more than 10% of the total population of contact maps. (A–C) illustrate the significant tetra-nucleosome contact patterns observed for Lin28A, while (D–F) depict the corresponding patterns for HoxA13.

Blob dynamics

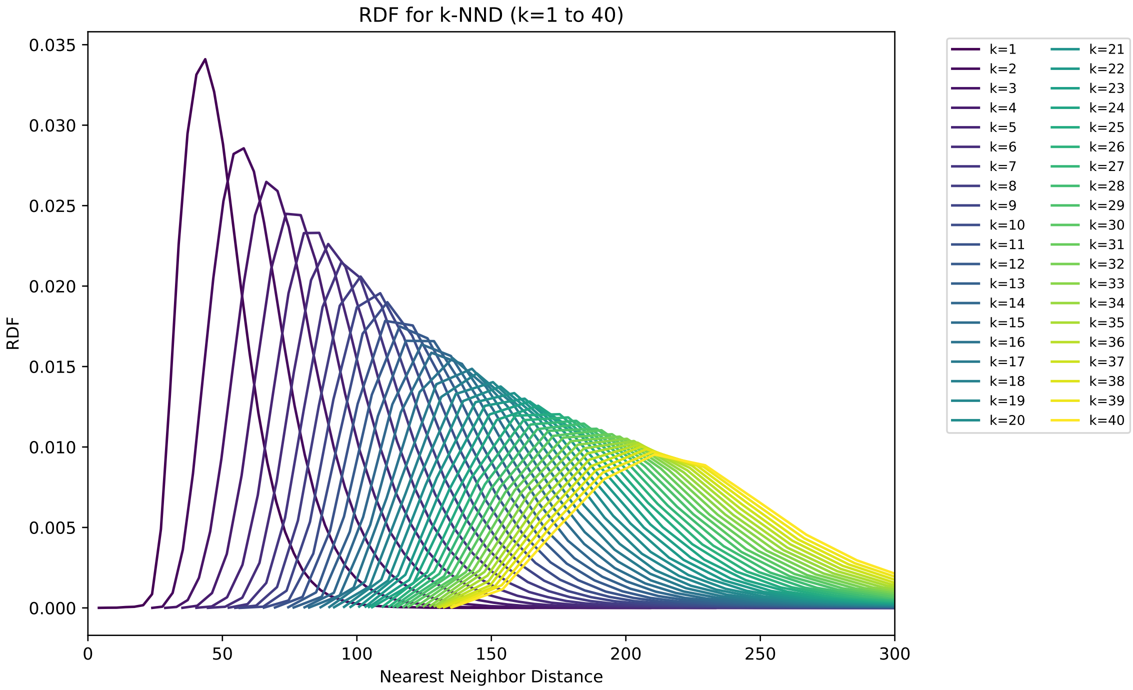

Supplementary Figure 13. Radial distribution of kth nearest neighbour distance of blobs for Nanog gene loci. Here, we show the nearest 1st to 40th neighbours distribution.

To understand the behaviour of the blobs, we further analyze the conformations obtained during the simulation. We calculate the Nearest Neighbor Distance (NND) of the blobs using the kth neighbouring distance (k = 1 to 40). The observed pattern closely resembles that reported by Barth et al.[12].

To further explore the blobs's dynamics, we compute the flow magnitude of blobs, which quantifies their movement between consecutive time steps. Additionally, we examine the variation in distances between different-sized blobs by calculating the radial distribution of surface area. We then analyze the spatial-temporal auto- and cross-correlation between surface area, NND, and flow magnitude to understand their interdependencies.

The autocorrelation of surface area indicates a high correlation at time lag zero, which quickly diminishes by time lag 4 and continues to decrease over a 40-unit time lag. This suggests that the surface area of blobs has minimal memory of its previous states. Spatially, the effect of surface area diminishes beyond 250 nm, whereas NND and flow magnitude exhibit some dependence on spatial lag, though the effect remains weak. The temporal analysis reveals that both NND and flow magnitude retain memory from previous time steps, persisting longer than surface area.

The spatial-temporal autocorrelation of flow magnitude closely mirrors that of NND, and their cross-correlation further supports this similarity, indicating that blob movement is more pronounced in regions where NND is higher. This suggests that spatially dispersed blobs exhibit greater movement than those that are more compact. Furthermore, the spatial-temporal cross-correlation between surface area and flow magnitude reveals that at a 250 nm spatial lag, blob motion has the greatest impact on blob size. This implies that the dynamics of blobs separated by approximately 250 nm influence surface area the most, with some dependency on previous time steps. However, as the distance decreases or exceeds 250 nm, this effect diminishes, along with its temporal memory.

Finally, the autocorrelation of surface area suggests that up to a 250 nm spatial lag, the surface area of one blob can influence that of others. However, beyond this range, the effect decreases, while flow magnitude—representing blob dynamics—plays a dominant role in determining blob size. Notably, at 250 nm, the influence of flow magnitude is most pronounced, but it weakens as spatial lag increases.

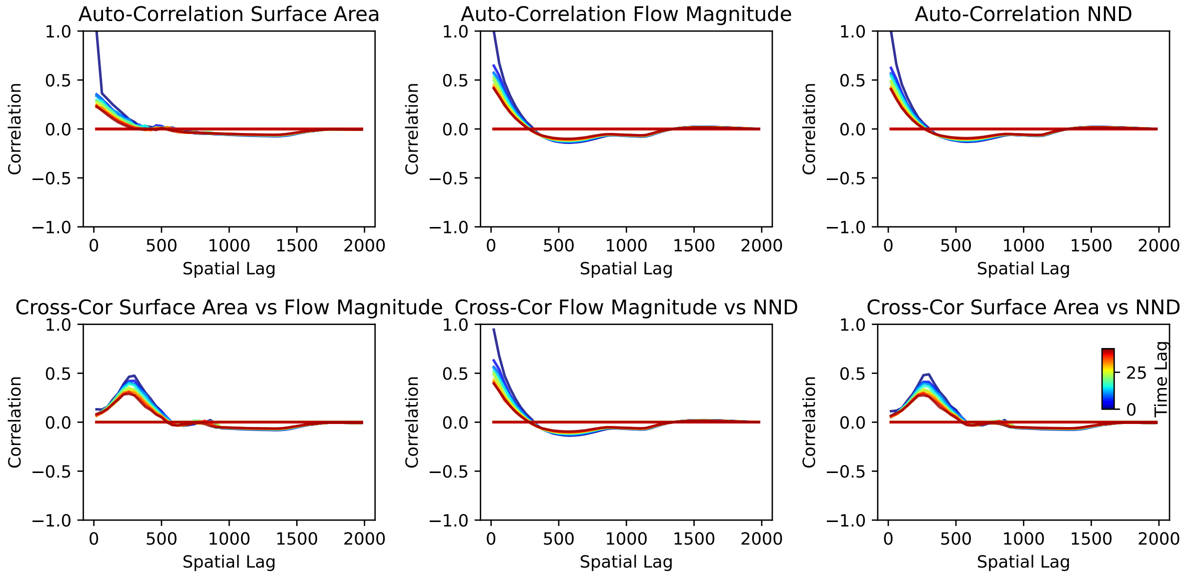

Supplementary Figure 14. Spatial-temporal auto and cross correlation between flow magnitude, NND and surface area of blobs for Nanog gene loci.

.

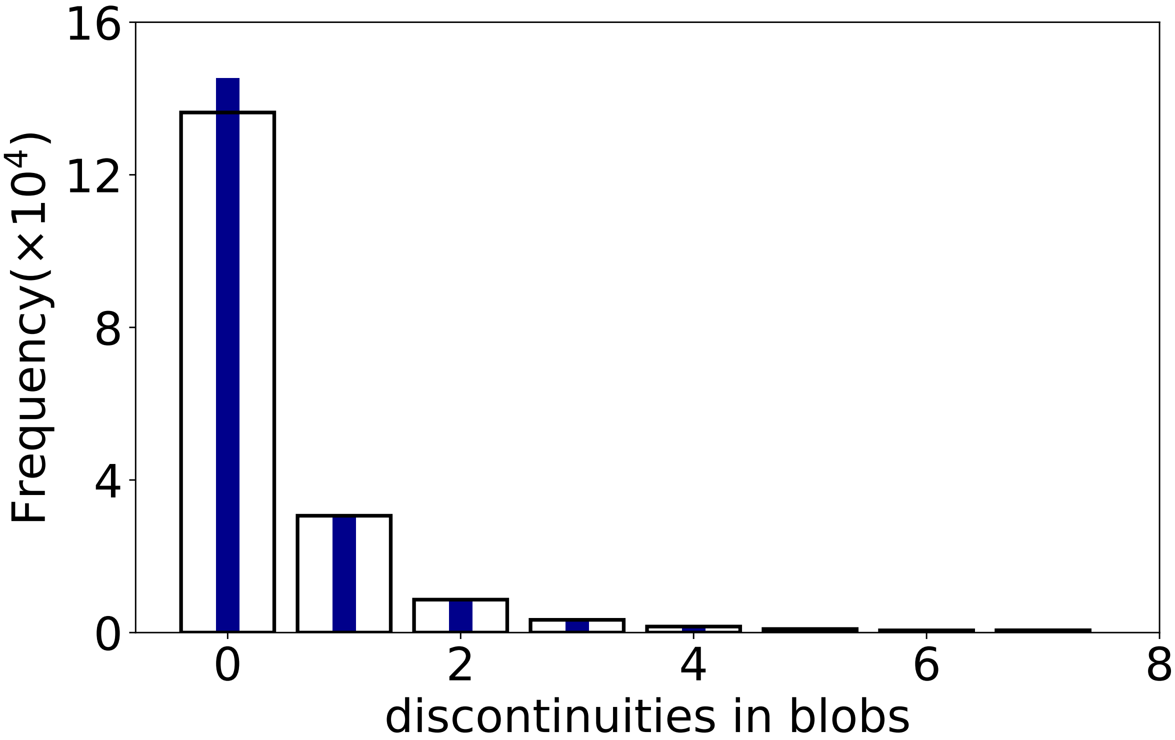

Supplementary Figure 15. Nuclesomes come together and form a blob. A sequential genomic segment forming a blob includes the sequential nucleosomes. But when distant genomic loci come closer and form a blob it can include N multiple sequential nucleosomes. These N multiple sequential nucleosomes suggest an N-1 break between sequential nucleosomes. The plot gives the histogram of breaks in the blobs and suggests that the blobs favour fewer breaks. The blue colour shows the histogram for the Nanog and the black border line shows the histogram for HoxB4.

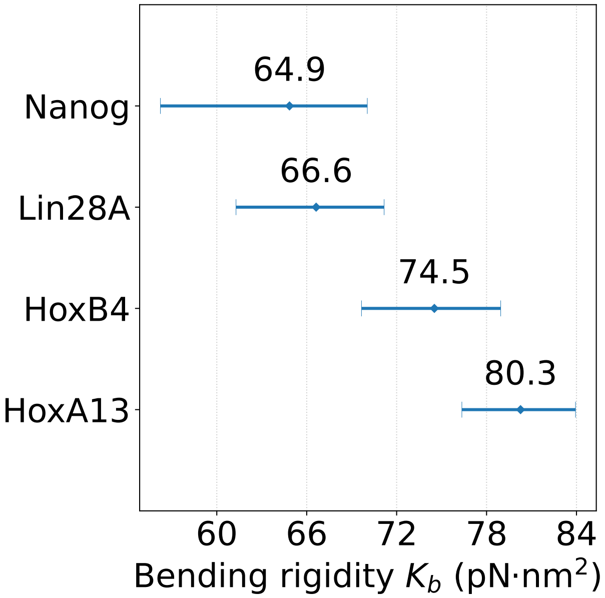

Supplementary Figure 16. Effective bending rigidity (K_b) estimates for Nanog, Lin28A, HoxB4, and HoxA13 loci using global tangent–tangent correlation methods. Data ranges are shown by a line having a center point as the mean value of the distribution. It suggests a similar pattern, we see in the main text Fig. 9A, where inactive loci (HoxA13, HoxB4) are stiffer than active loci (Nanog, Lin28A).

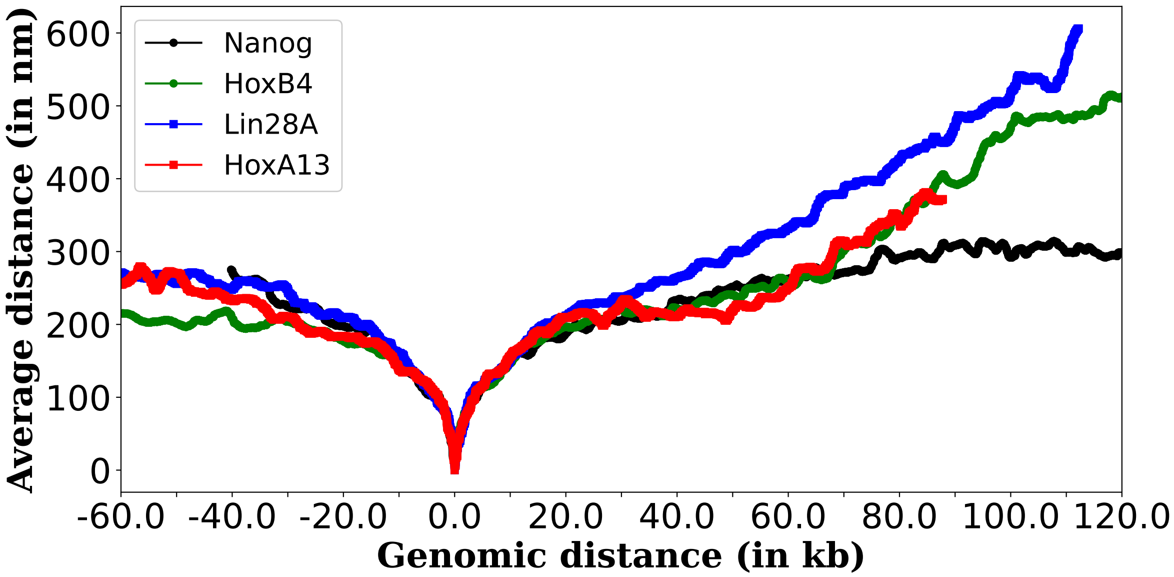

Supplementary Figure 17. Gene loci and their impact on long-range genomic interactions. The figure illustrates how differences in bending rigidity(Supplementary Figure 16) influence long-range genomic interactions by estimating the distances of genomic segments from the transcription start sites (TSS) of all gene loci. The relatively larger distance of downstream genomic segments from the TSS in HoxB4 and HoxA13 suggests a reduced likelihood of long-range enhancer-promoter interactions, in contrast to the Nanog locus, where such interactions are more probable. The Lin28A gene locus exhibits a contradictory behaviour compared to the Nanog locus. Their different role in the cell could be the reason, where Nanog is a homeobox transcription factor and Lin28A is an RNA-binding protein.

Supplementary References

1. Kadam, S. *et al.* Predicting scale-dependent chromatin polymer properties from systematic coarse-graining. *Nat. Commun.* **14**, 4108 (2023).
2. Chen, K. & others. Danpos: dynamic analysis of nucleosome position and occupancy by sequencing. *Genome Res.* **23**, 341–351 (2013).
3. Wiese, O. *et al.* Nucleosome positions alone can be used to predict domains in yeast chromosomes. *Proc. Natl. Acad. Sci. USA* **116**, 17307–17315 (2019).
4. Qi, Y., Zhang, B. *et al.* Predicting three-dimensional genome organization with chromatin states. *PLoS Comput. Biol.* **15**, e1007024 (2019).
5. Chiariello, A. M., Abraham, A. *et al.* Multiscale modelling of chromatin 4d organization in sars-cov-2 infected cells. *Nat. Commun.* **15**, 1–12 (2024).
6. Krietenstein, N. *et al.* Ultrastructural details of mammalian chromosome architecture. *Mol Cell* **78**, 554–565.e7 (2020).
7. Cremer, T., Cremer, M. *et al.* The 4d nucleome: Evidence for a dynamic nuclear landscape based on co-aligned active and inactive nuclear compartments. *FEBS Lett.* **589**, 2905–2913 (2015).
8. Ernst, J. *et al.* *ChromHMM* : automating chromatin-state discovery and characterization. *Nat Methods* **9**, 215–216 (2012).
9. Wolff, J., Bhardwaj, V. *et al.* Galaxy hicexplorer: a web server for reproducible hi-c data analysis, quality control and visualization. *Nucleic Acids Res.* **46**, W11–W16 (2018).
10. Wolff, J., Backofen, R. *et al.* Loop detection using hi-c data with hicexplorer. *Gigascience* **11**, giac061 (2022).
11. Hsieh, T. *et al.* Mapping Nucleosome Resolution Chromosome Folding in Yeast by Micro-C. *Cell* **162**, 108–119 (2015).
12. Barth, R. *et al.* Coupling chromatin structure and dynamics by live super-resolution imaging. *Sci. Adv.* **6**, eaaz2196 (2020).
